# Persistent Mullerian Duct Syndrome: a rare entity with a rare presentation in need of multidisciplinary management

**DOI:** 10.1590/S1677-5538.IBJU.2016.0225

**Published:** 2016

**Authors:** Lin Da Aw, Murizah M. Zain, Sandro C. Esteves, Peter Humaidan

**Affiliations:** 1 Department of Obstetrics & Gynaecology, Hospital Sultanah Bahiyah, Kedah Darul Aman, Malaysia; 2 Fertility Clinic, Skive Regional Hospital, Denmark; 3 Androfert, Andrology & Human Reproduction Clinic, Referral Center for Male Reproduction, Campinas, Brazil; 4 Faculty of Health, Aarhus University, Denmark

**Keywords:** Persistent Müllerian Duct Syndrome [Supplementary Concept], Disorders of Sex Development, Hydrocolpos

## Abstract

**Main findings:**

A typical male looking adolescent with a legal female gender assignment presented with haematuria. Investigations led to the diagnosis of Persistent Mullerian Duct Syndrome. The condition is indeed a rare entity that needs a multidisciplinary team management.

**Case hypothesis:**

A case of Persistent Mullerian Duct Syndrome undiagnosed at birth because karyotyping was defaulted, thus resulting in a significant impact on the legal gender assignment and psychosocial aspects.

**Promising future implications:**

The reporting of this case is important to create awareness due to its rarity coupled with the rare presentation with hematuria as a possible masquerade to menstruation. There were not only medical implications, but also psychosocial and legal connotations requiring a holistic multidisciplinary management.

## INTRODUCTION

Persistent Mullerian Duct Syndrome (PMDS) is a very rare condition with less than 300 cases described in the literature (1, 2). This rare entity showcases adolescents who are phenotypically males with 46,XY karyotypes. However, such individuals harbor internal female reproductive organs which are Mullerian derivatives, rendered by a defect in either genes coding for the Mullerian inhibiting substance (MIS) / anti-Mullerian Hormone (AMH) or the AMH receptor (2), ultimately leading to failure of regression of Mullerian ducts (3).

### Scenario

A 14-year-old adolescent with typical male physical appearance, legal gender assigned as female, presented to the emergency department with hematuria. Abdominal ultrasound showed normal sized kidneys with a few small nephrolithiasis of 3–4mm in size. There was no hydronephrosis and the urinary bladder looked normal. However, there was a cystic lesion located postero-superior to the urinary bladder, suspected to be a hydrocolpos. By definition, hydrocolpos refers to the distension of the vagina caused by accumulation of fluid due to congenital vaginal obstruction. The patient was subsequently referred to the gynecology department for assessment of hydrocolpos.

On further questioning, the patient had intermittent hematuria, however, denied cyclical abdominal pain or backache. The history from the mother revealed a patient who was born prematurely at 32 week's gestation from a non-consanguineous union and who had ambiguous genitalia at birth with the absence of both testes. The patient was referred to a university hospital for further investigation, from which ultrasound of the pelvis detected a uterus-like structure. The baby was given appointment to have karyotyping performed, however the mother had defaulted the investigation as the child's father was terminally ill. With financial restriction, logistic problems, raising the child single-handedly and without any ill intention, the mother had gender assigned this child as a female with the finding of a uterus-like structure.

The child was initially placed in a normal primary school but was unable to follow school routine in view of his mental capacity that is below the average required by normal school; it was advised for her to be admitted to a school for children with special needs. The patient excelled in sports and had the strength of a male–even preferred male peer groups and male attire.

A physical examination revealed an adolescent with a typical male phenotype, height within 50^th^ centile of boys in accordance to race, age and measured height. The patient had facial acne vulgaris, a coarse voice and male pattern hair distribution. Further examination revealed a normal abdomen and inguinal region. An examination of the genitals showed a well-formed penis, with a urethral opening at the ventral aspect of the penile root. There was absence of testes bilaterally in the scrotum; however, no vaginal opening was seen.

Karyotyping showed – 46,XY with SRY gene present. Follicle Stimulating Hormone: 44.09U/L (normal: 0.05–9.60U/L), Luteinizing Hormone: 15.19U/L (0.08–20.62U/L), Total Testosterone: 4.34nmol/L (normal: 10.07–38.76nmol/L), Estradiol: 24.5pmol/L (follicular: 77–921pmol/L, luteal: 77–1145 pmol/L), Aldosterone: 530pmol/L (>11 years old: <582.6pmol/L), Serum Dehydroepiandrosterone: 94ug/dL (20–49 years old, female: 32–380ug/dL, male: 94–640ug/L), Cortisol: 201nmol/L (normal, 9am: 64–536nmol/L), beta HCG: 6.5IU (<5.0IU), alfa fetoprotein 0.9IU/mL (normal: 0–5.8IU/mL), Free Thyroxine: 14.71pmol/L (9.14–23.8pmol/L), Thyroid Stimulating Hormone: 1.66mIU/L (0.27–4.2mIU/L), Insulin-like Growth Factor-1: 403ug/L (normal for 13–15 years old: 142–525ug/L).

**Figure 1:**
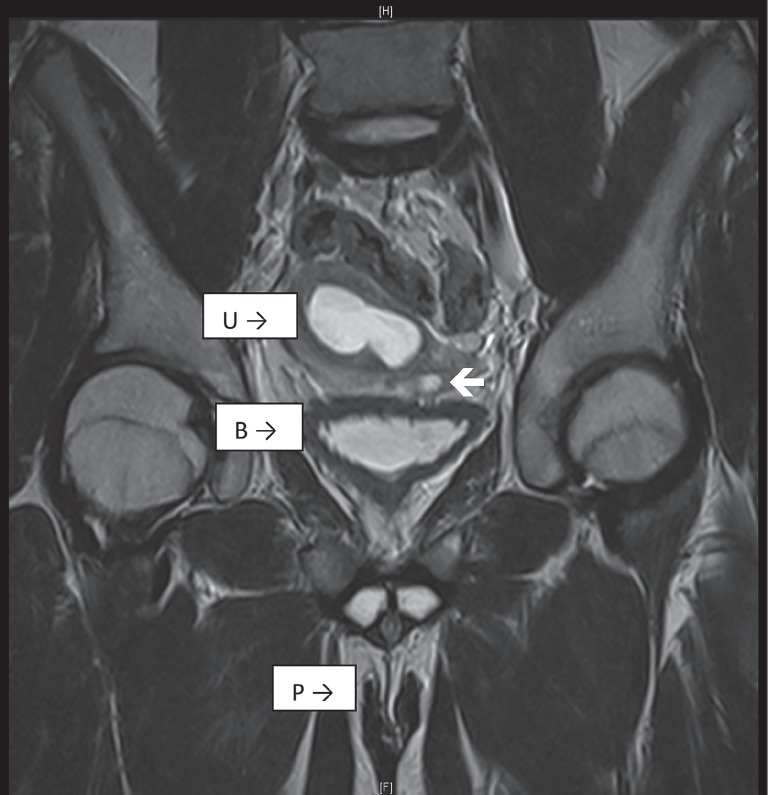
MRI of the pelvis and abdomen of the patient, showing the bladder (B), a single cervix and uterus (U), and two tubular structures at the usual place of corpus cavernosum (P). Structures suggesting the ovaries are not seen. The uterus has two horns, however it has flat fundus which is in favor of incomplete septate uterus. An oval intermediate signal intensity at left hemipelvis, medial to external iliac vessels, is suggestive of a testes. It measured 1.2cm × 1.2cm × 2.3cm.

**Figure 2:**
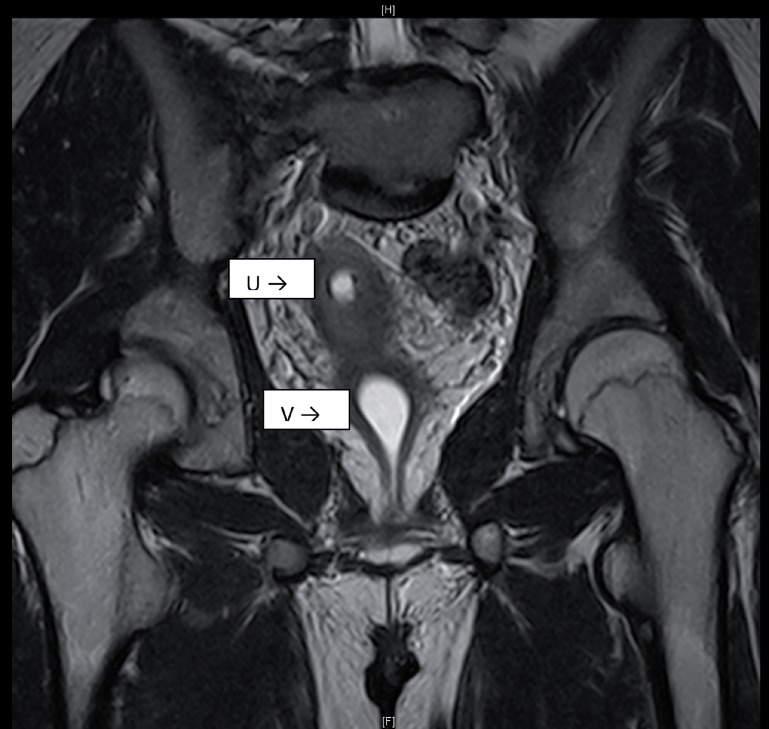
MRI of the pelvis, showing the uterus (U) and the hydrocolpos (V).

MRI of the abdomen and pelvis showed a single cervix, uterus and hydrocolpos (Figures 1 and 2). The uterus had two horns with a flat fundus. There were no definite structures to suggest ovaries. However, there was an oval structure at the left hemi pelvis, medial to the external iliac vessels, suggestive of a testis, measuring 1.2x1.2x2.3cc. A cysto-genitoscopy noted an opening at the posterior and distal part of the bladder neck, enabling the scope to pass through, finding blood clots in a space possibly representing the vagina. A cervix was not identified. The urethra, bladder and urethral opening were otherwise normal.

Diagnostic abdominal laparoscopy revealed a tubular structure in the left pelvic region, which might represent a streak gonad, with another tubular structure situated superiorly, a possible Wolffian remnant. A bicornuate uterus with a more prominent right horn was identified with a long tubular structure connected to the right uterine horn that represented a fallopian tube. No gonad was identified at the distal end of the fallopian tube. A rounded structure enlarged to the superior of the uterus was characterized as a possible hydrocolpos.

A diagnosis of PMDS with one identified abdominal testis was made. Subsequently, the child and the mother requested for a legal change in gender assignment due to the problems faced. The patient was referred to joined management with a urologist, a pediatric surgeon and a pediatric adolescent psychiatrist. An application for legal gender change was filed and the child was subsequently planned for hysterectomy with the excision of the hydrocolpos and the streak gonad.

## DISCUSSION

The mechanism of PMDS is likely to be multi-factorial, although there have been reports of consanguinity. An autosomal recessive inheritance seems to affect the majority (85%) of cases (2); however, the origin of the remaining 15% of cases remains unknown (3). Nonetheless, the case reported did not arrive from a consanguineous union (2).

The condition exhibits a wide variation in the age of presentation, being reported in a man with 77 years of age (1). It may also be undiagnosed, as in some cases the Mullerian derivatives do not give rise to any problems to the bearer. A list of the PMDS cases reported in the literature in the last five years is provided in Table-1 (4–26). And up to date, there are no reported cases of association with mental abnormality with PMDS. Therefore, we believe that the patient has coincidental mental handicap.

**Table 1:** Case reports of Persistent Mullerian Duct Syndrome (PMDS) in the last five years*

First author and year (ref. number)	Country	Patient characteristics
Tosur 2015 (4)	United States	A genetic male with PMDS and AMH deficiency associated with distal monosomy 10q.
Modi 2015 (5)	India	An adult male with bilateral cryptorchidism and a pelvic mass, who presented with acute urinary retention, and was diagnosed with a seminoma of the right testis, intratubular germ cell neoplasia of the left testis with the presence of Mullerian remnants.
Yamada 2015 (6)	Japan	A 42-year-old man with testicular seminoma and transverse testicular ectopia.
Kovachev 2014 (7)	Bulgaria	A 46-year-old man with a history of cryptorchidism and orchidopexy and father of two children presenting with an intra-abdominal mass.
Telli 2014 (8)	Turkey	A patient with testicular ectopia.
Morikawa 2014 (9)	Japan	A 1-year-old child presented with bilateral cryptorchidism and normal male external genitalia. A laparoscopic surgery revealed a uterus and fallopian tubes.
Wei 2014 (10)	Taiwan	Three patients with bilateral undescended testis and Mullerian duct remants.
Alp 2014 (11)	Turkey	A 28-year-old male diagnosed with undescended left testis and infertility (azoospermia).
Wu 2014 (12)	United States	An infant born with hypospadias and no palpable gonads was diagnosed with PMDS based on history, physical examination, laboratory testing, and radiologic imaging. A robot-assisted laparoscopic hysterectomy, right gonadal biopsy, and bilateral orchidopexy were performed.
Farag 2013 (13)	United Kingdom	A young male with a left sided inguinal hernia in which the sac contained both testes and uterus.
Beatty 2013 (14)	United States	A 50-year-old man with a history of hernia repair and vanished testes presenting with abdominal pain, who was diagnosed with an abdominal mass shown to be an uterus and ovary.
Gupta 2013 (15)	India	A 23-year-old man presented with a left-sided reducible inguinal hernia and undescended testis in an empty ill-developed right hemiscrotum. Semen analysis revealed azoospermia and laparoscopy demonstrated a uterus, fallopian tube and a testis.
Wongprasert 2013 (16)	United States	A 2-week-old newborn presented with bilateral cryptorchidism and normal male external genitalia. At age 1 year and 4 months, he underwent laparoscopic surgery, which revealed a uterus and fallopian tubes. A unique homozygous T to G base substitution was found at position 2219, near the middle of the exon 5, changing codon CTG to CGG in anti-Mullerian hormone (AMH) gene. Both parents are heterozygous for the mutation.
Østergren 2013 (17)	Denmark	One patient with unilateral undescended testes and PMSD found during laparoscopy.
Ju 2013 (18)	China	Three cases of Chinese patients with PMDS associated with testicular ectopia, which were subjected to orchidopexy.
Salehi 2012 (19)	United States	Description of 8 cases diagnosed in California.
Keukens 2012 (20)	Netherlands	A 12-year-old boy with cryptorchidism and PMSD diagnosed during laparoscopy.
van der Zwan 2012 (21)	Netherlands	A PMSD patient diagnosed with a novel homozygous missense mutation in the AMH gene (single nucleotide insertion (C) at position 208 Biopsy of both gonads revealed that germ cells were present in an irregular distribution, but the absence of OCT3/4, PLAP and c-KIT expression indicated physiological maturation.
Bassani 2012 (22)	Italy	A patient with PMDS and ITGCNU.
Farikullah 2012 (23)	United Kingdom	Eleven cases with PMDS and malignancy of the Müllerian remnants were reported: five males presented with bilateral undescended testes and three had unilateral undescended testis.
Chattopadhyay 2011 (24)	India	A phenotypically normal looking male presenting with irreducible left sided inguinal hernia, which on exploration revealed uterus, fallopian tubes and testis.
Demir 2011 (25)	Turkey	A 37-year-old man with history bilateral cryptorchidism, erectile dysfunction and infertility; diagnostic laparoscopy confirmed the presence of intra-abdominal testes and PMDS.
Kaul 2011 (26)	United States	An adult male who had a unique combination of both transverse testicular ectopia and PMDS presenting as an incarcerated inguinal hernia.

* Pubmed; articles in English.

**AMH** = anti-Mullerian hormone; **ITGCNU** = intratubular germ-cell neoplasia of unclassified type.

This child had previously presented with hematuria, without which he might have remained silent in his anguished female-assigned role. Ultrasound of kidney, ureter and urinary bladder was performed, from which hydrocolpos was detected. Hematuria, here, could be renal of origin or a mixture of urine with menstrual blood from a possible functioning uterus. This patient, however, did not have other associated symptoms of colicky pain or dysuria to suggest renal origin. In a study involving 292 children, about 15% of the subjects with renal calyceal microlithiasis of <3mm presented with hematuria, suggesting the minute size of calculi and their ability to pass through the urinary tract do not aggravate crystaluria (27). A previous review on nephrolithiasis in children revealed that about 30% of children present with gross hematuria; however, only 4 out of 50 children with the presence of an average of 2.3mm urolithiasis have clinical symptoms leading to diagnosis of distal ureteral stones (28).

The serum hormonal profile showed a corresponding picture of testicular failure in which he had a high FSH level and a low testosterone level from the hypo-production of the streak male gonad. In the management of young children with undescended testes, serum AMH measurement can be beneficial in assessing the gonadal function, reflecting the normal development of male genitals. A measurable AMH in a boy who presented with bilateral cryptorchidism is predictive of undescended testes, while undetected AMH is suggestive of anorchia or the presence of ovaries as in cases of pure gonadal dysgenesis or female pseudohermaphroditism (29, 30). Unfortunately, AMH was not measured due to unbearable cost to the family.

The testicular descent indeed is a very complex process influenced by different hormones. Insulin-like hormone 3 (INSL3) is a Leydig cell hormone, which controls the initial gubernacular enlargement (31). Androgens control the inguino-scrotal migration, possibly following signaling from the mammary line and the genito-femoral nerve (31). Although the abnormal long gubernacular cord in cases of PMDS remains unexplained, it is possible that this feature relates to lack of decrease in gubernaculum size that coincides with the complete descent of the testicle-a phenomenon that is androgen-dependent (32). It has been shown that androgen deficiency is associated with failed regression of the gubernaculum and cryptorchidism (33). Despite recent discoveries concerning testicular migration, there are more complex processes that need further understanding. To some, orchidopexy is recommended in the first year of life in view of increasing reports on the formation of stem cells for spermatogenesis to occur between 3 and 9 months of life. Although the aim of surgery is to restore this process and prevent abnormal gonocyte maturation, this is yet to be further confirmed. On the other hand, there is the acquired type of undescended testis, usually presenting later in childhood (34). It occurs when the spermatic cord elongation fails between birth and puberty, so that the initially descended testis is left behind as the scrotum moves further from the groin as the boy enlarges. The prognosis for this variant seems to be better than congenital undescended testis because gonocyte development in the first year should be normal while the testis is still in the scrotum. Currently, orchidopexy and hormonal treatment are valid treatment options for acquired undescended testis; however, many prefer the scrotal surgery when the testis is not residing spontaneously in the scrotum (31).

Patients with PMDS and gonadal dysgenesis who have Y chromosome material are at increased risk for developing germ cell tumors such as gonadoblastoma or carcinoma in situ, with the potential for malignant transformation to dysgerminoma or seminoma, respectively. The prevalence of germ cell tumors in such patients varies from 15–40% (35). The evidence for routine screening with serum tumor markers is equivocal, albeit mandatory if the patient has a gonadal mass on pre-operative imaging (36). If tumor markers are positive, a staged surgical procedure (laparotomy instead of laparoscopy) is indicated. In patients with streak intra-abdominal gonads, gonadal biopsy has no role, as these patients ultimately require gonadectomy to prevent development of a malignancy (36). Recent literature advocates simultaneous hysterectomy and orchidopexy (2), with the laparoscopic approach gaining popularity in recent years (37, 38). The question of reversion of virilization after gonadectomy was not an option for our patient; hence, life-long testosterone replacement therapy was advised.

The request for legal change of gender assignment is valid; however, it might have been avoided if karyotyping had been performed earlier at birth, with the discovery of ambiguous genitalia with bilateral undescended testes. Given the circumstances that surrounded the family's condition at that particular moment, that is the serious illness of one parent, financial restrictions and logistic problems rendered the default to treatment. The failure to understand the seriousness of the problem and its future implications presumably has led to the patient's mistaken identity. Therefore, it was crucial to involve a pediatric/adolescent psychiatrist for the evaluation of the psychological impact of being raised as a female and whether a gender re-assignment would have any significant negative impact on the patient.

Gender assignment is a controversial issue in cases of disorders of sexual differentiation (DSD). Based on no documented report of malignant transformation in fully descended testes, some advocate male gender assignment to significantly virilized patients with completely bilateral descended testes. Another school of experts prefers female gender assignment in patients who have a uterus and a vagina and who are under-virilized and are markedly short in height (39). In our case, however, it was interesting to find a completely virilized adolescent with low testosterone. Nevertheless, a possible explanation for this finding is that such patients grow up with lower than normal levels of testosterone and hence their threshold of normal testosterone required for virilization is lower, as seen in other genetic conditions in which virilization is achieved despite having low testosterone levels (e.g. Klinefelter syndrome and 46,XX DSD).

Importantly, there were many issues that needed to be addressed in this patient; hence, several experts from various fields, including urologists, gynecologists, geneticists and psychiatrists, were organized to evaluate the patient. Thus, a comprehensive multidisciplinary team management is the recommended method of managing similar cases with rare disorders of sexual differentiation of complicated nature in the future 5–6, (40).

## CONCLUSIONS

A clinically challenging case of Persistent Mullerian Duct Syndrome is discussed that required a multidisciplinary team approach, delineating each and every problem with the aim to create awareness among health care providers of a rare entity.
